# Effect of Fufang Huangqi Decoction on the Gut Microbiota in Patients With Class I or II Myasthenia Gravis

**DOI:** 10.3389/fneur.2022.785040

**Published:** 2022-03-18

**Authors:** Yanghong Chen, Yi Lin, Caifeng Shan, Zhaoqing Li, Bo Xiao, Rencai He, Xueshi Huang, Zhanyou Wang, Jingsheng Zhang, Wenjun Qiao

**Affiliations:** ^1^The Affiliated Hospital of Liaoning University of Traditional Chinese Medicine, Liaoning Provincial Key Laboratory for Diagnosis and Treatment of Myasthenia Gravis, Liaoning University of Traditional Chinese Medicine, Shenyang, China; ^2^Department of General Surgery, The First People's Hospital of Shenyang, Shenyang, China; ^3^Zhejiang Jiuru Pharmaceutical Technology Co., Ltd., Hangzhou, China; ^4^Institute of Microbial Pharmaceuticals, College of Life and Health Sciences, Northeastern University, Shenyang, China; ^5^Institute of Health Sciences, Key Laboratory of Medical Cell Biology of Ministry of Education, China Medical University, Shenyang, China

**Keywords:** myasthenia gravis, gut microbiota, taxonomic markers, probiotics, Fufang Huangqi Decoction

## Abstract

**Objective:**

To investigate the effect of Fufang Huangqi Decoction on the gut microbiota in patients with class I or II myasthenia gravis (MG) and to explore the correlation between gut microbiota and MG (registration number, ChiCTR2100048367; registration website, http://www.chictr.org.cn/listbycreater.aspx; NCBI: SRP338707).

**Methods:**

In this study, microbial community composition and diversity analyses were carried out on fecal specimens from MG patients who did not take Fufang Huangqi Decoction (control group, *n* = 8) and those who took Fufang Huangqi Decoction and achieved remarkable alleviation of symptoms (medication group, *n* = 8). The abundance, diversity within and between habitats, taxonomic differences and corresponding discrimination markers of gut microbiota in the control group and medicated group were assessed.

**Results:**

Compared with the control group, the medicated group showed a significantly decreased abundance of Bacteroidetes (*P* < 0.05) and significantly increased abundance of Actinobacteria at the phylum level, a significantly decreased abundance of Bacteroidaceae (*P* < 0.05) and significantly increased abundance of Bifidobacteriaceae at the family level and a significantly decreased abundance of *Blautia* and *Bacteroides* (*P* < 0.05) and significantly increased abundance of *Bifidobacterium, Lactobacillus* and *Roseburia* at the genus level. Compared to the control group, the medicated group had decreased abundance, diversity, and genetic diversity of the communities and increased coverage, but the differences were not significant (*P* > 0.05); the markers that differed significantly between communities at the genus level and influenced the differences between groups were *Blautia, Bacteroides, Bifidobacterium* and *Lactobacillus*.

**Conclusions:**

MG patients have obvious gut microbiota-associated metabolic disorders. Fufang Huangqi Decoction regulates the gut microbiota in patients with class I or II MG by reducing the abundance of *Blautia* and *Bacteroides* and increasing the abundance of *Bifidobacterium* and *Lactobacillus*. The correlation between gut microbiota and MG may be related to cell-mediated immunity.

## Introduction

Myasthenia gravis (MG) is an acquired autoimmune disease mediated by autoantibodies and characterized by neuromuscular junction transmission disorders. Its clinical features are mainly weakness of the ocular muscles (incidence of 85%) and partial or systemic weakness of skeletal muscles; respiratory muscle paralysis that can lead to dyspnea may occur in severe cases. The incidence and prevalence of MG are gradually increasing, and the incidence has increased most notably among the elderly (1). The pathogenesis of MG is still unclear. Research on the pathogenesis of MG has mainly focused on the following 3 aspects: mechanisms of cell-mediated immunity, the abnormal expression of multiple pathogenic antibodies and environmental factors. In cell-mediated immunity, Th1, Th17 and regulatory T cells (Tregs), differentiated as CD4+ T cells, play important roles in the occurrence and development of MG. A large number of studies have shown that MG is associated with multiple pathogenic antibodies, including anti-muscle-specific tyrosine kinase (MuSK) antibodies, anti-low-density lipoprotein receptor-related protein 4 (LRP4) antibodies and anti-acetylcholinesterase (AChE) antibodies (2). In addition, some studies have proposed that the pathogenesis of MG is also related to environmental factors such as microorganisms (3). At present, the treatments for MG are generally divided into symptomatic treatment and immunotherapy. Acetylcholinesterase inhibitors (AChEIs) are the most common symptomatic treatment, and pyridostigmine bromide is currently used as a long-term oral drug for relieving symptoms in MG patients. Regarding immunotherapy, glucocorticosteroids, methotrexate, azathioprine, cyclosporine A, cyclophosphamide, tacrolimus and rituximab are widely used, but they have many adverse reactions (4), such as nausea, diarrhea, liver and kidney damage and osteoporosis. The long-term administration of hormones, AChEIs and immunosuppressants may result in drug resistance and relapse or even aggravated conditions after drug withdrawal. Although there are many medications indicated for the treatment of MG, gut microbiota-regulating drugs have not yet been applied for the clinical treatment of MG.

The gut microbiota has attracted much attention in recent years, and a healthy gut microbiota is mainly dominated by Firmicutes and Bacteroidetes, followed by Actinobacteria (5). As the first internal defense against pathogens and toxins, the gut microbiota in the normal human body protects the body from diseases in conjunction with the host immune system. The gut microbiota is very important for the normal development and function of FOXP3 Tregs. The protective effect of microorganisms can be regulated by downregulating proinflammatory cytokines (IL-8, IL-12, and IL-23) and upregulating anti-inflammatory cytokines (IL-10) produced by Tregs (6). The gut microbiota is responsible for important metabolic and signaling functions (7) and has complex and extensive effects on the human body. The gut microbiota is closely related to gastrointestinal diseases (8), cardiovascular and cerebrovascular diseases (9), metabolic diseases (10), autoimmune diseases (11) and mental diseases (12). Changes in the gut microbiome have also been associated with the occurrence of diseases caused by the “microbiota-gut-brain” (MGB) axis, including stroke, epilepsy, Parkinson's disease, depression and schizophrenia (13). An imbalance in the gut microbiota has been observed in MG patients. Compared with those in a healthy control group, the relative proportions of *Bacteroides* and *Desulfovibrio*were higher, and the relative proportions of Verrucomicrobiaceae and Bifidobacteriaceae were lower (14). Therefore, it is possible that adjusting the balance of the gut microbiota could an important approach for treating MG. Traditional Chinese medicine has unique treatment methods for MG resulting in remarkable treatment efficacy for this rare autoimmune disease. In the clinical treatment of MG, Fufang Huangqi Decoction takes effect within 4 weeks, considerably improving symptoms. After the use of Fufang Huangqi Decoction for 3–5 years, symptoms stabilize, and the dose of Western medicine can be lowered to reduce side effects (15). However, the mechanism by which Fufang Huangqi Decoction improved MG is still unclear. In this study, we analyzed the gut microbiota in patients with class I or II MG who took Fufang Huangqi Decoction and achieved remarkable symptom alleviation and those who did not take the medicine, and we explored the effect of Fufang Huangqi Decoction on the gut microbiota in patients with class I or II MG.

## Materials and Methods

### General Data

In this study, 16 patients with class I or II MG who visited the Affiliated Hospital of Liaoning University of Traditional Chinese Medicine from March 2021 to April 2021 were randomly selected. Patients who did not take Fufang Huangqi Decoction (*n* = 8) and those who took Fufang Huangqi Decoction and achieved obvious symptom alleviation (*n* = 8) served as the control group and the medicated group, respectively. There was no significant difference in age, sex and course of disease between the 2 groups (*P* > 0.05) (See Table 1). The study was approved by the ethics review committee of the Affiliated Hospital of Liaoning University of Traditional Chinese Medicine, and all patients signed an informed consent form.

**Table 1:** Comparison of general data between the control group and the medicated group.

**Group**	**All (*n =* 16)**	**Male/female**	**Age (months)**	**Course of disease (months)**
Control group	8	4/4	52.13 ± 14.50	9.63 ± 12.28
Medication group	8	7/1	53.88 ± 16.15	21.88 ± 12.41
P		0.134	0.346	0.883

### Inclusion and Exclusion Criteria

The inclusion criteria were as follows: (1) met the clinical diagnostic criteria of MG (16, 17); (2) diagnosis of class I or II MG based on the modified Osserman classification (18); (3) remarkable alleviation of symptoms after taking Fufang Huangqi Decoction (absolute and relative scores >25% based on the clinical absolute and relative scoring system proposed by Xianhao Xu) (19); (4) no administration of immunosuppressants, hormones or other drugs that may affect this observational study 3 months prior to taking Fufang Huangqi Decoction; (5) no thymic abnormality, thymoma or thymic hyperplasia; and (6) informed consent and signed informed consent forms from patients and their families.

The exclusion criteria were as follows: (1) ongoing MG-related treatments that might affect the efficacy assessment; (2) class III, IV or V MG and MG crisis; (3) other autoimmune diseases, such as rheumatoid arthritis, vitiligo and systemic lupus erythematosus; (4) pregnancy or lactation, allergic constitution, critically ill or late stage of disease; (5) severe heart, liver and kidney diseases; (6) patients who did not cooperate with the study, were unwilling to undergo study assessments or failed to cooperate because of mental illness; and (7) failure to carry out follow-up visits.

### Fecal Collection and DNA Extraction

Fresh feces (approximately 5 g) were collected from patients in the control group (after the first visit, without taking Fufang Huangqi Decoction) and the medicated group (at the time of remarkable alleviation of symptoms after taking Fufang Huangqi Decoction); the samples were immediately put into sterile freezable tubes, frozen quickly in liquid nitrogen and stored in a cryogenic freezer at −80 °C. OMEGA M5635-02 extraction kits were used by Norson Biotechnology Co., Ltd. to extract the DNA of fecal microbiota for microbial diversity sequencing and sequence processing.

### Microbial Diversity Sequencing Method

Approximately 500 mg of sample was added directly into a 2-ml centrifuge tube. Then, 500 mg of glass beads and 0.8 ml of SLX Mlus Buffer was added to the tube. The sample was shaken for 7 min at 25 Hz in a tissue grinder, after which 80 μl of Buffer DS was added to the tube, followed by shaking to mix thoroughly. Next, the sample was incubated at 90 °C for 10 min, and the tube was inverted once or twice to mix the sample during incubation. Then, the sample was centrifuged at room temperature for 5 min at 13,000 × g (12,000 rpm). A total of 600 μl of supernatant was transferred from the tube to a new 2-ml centrifuge tube, to which 200 μl of Buffer SP2 was added. The tube was gently shaken to manually mix the sample. A total of 100 μl of HTR reagent was added to the tube, followed by light shaking to mix the sample evenly. The sample was placed in an ice bath for 5 min and centrifuged at 13,000 × g (12,000 rpm) for 5 min. Afterwards, 400 μl of supernatant was carefully transferred to a 96-deep-well plate, to which 40 μl of magnetic beads and 450 μl of binding buffer was added and mixed well; the plate sat at room temperature for 2 min. The obtained sample was then purified with a nucleic acid purifier and subjected to PCR amplification after determining the concentration of DNA in each sample. The highly variable V3V4 region of the bacterial 16S rRNA gene (approximately 468 bp) was selected for PCR amplification and sequencing; the specific primers for the bacterial 16S rDNA V3V4 region were as follows: 338F (5′-barcode+ACTCCTACGGGAGGCAGCA-3′) and 806R (5′-GGACTACHVGGGTWTCTAAT-3′). The amplification products were subjected to 2% agarose gel electrophoresis. The target fragments were cut out and then recovered using an Axygen gel recovery kit. The PCR products were quantified using a microplate reader (BioTek, FLx800) and a Quant-iT PicoGreen dsDNA Assay Kit and were then mixed based on the concentration needed to construct a library. A TruSeq Nano DNA LT Library Prep Kit (Illumina) was used to construct the library. After a quality inspection and quantification of the library, paired-end sequencing (2 × 250 bp) was conducted for the qualified library on a MiSeq machine using a MiSeq Reagent Kit V3 (600 cycles). The library used for sequencing was diluted to 2 nM, and samples were mixed proportionally based on the required data size. For sequencing, the mixed library was denatured into single strands using 0.1 N NaOH.

### Sequence Processing

QIIME2 (April 2019) software was used for primer removal, quality filtering, denoising, splicing and chimera removal by the DADA2 method (see Supplementary Ref 1). The characteristic sequence equivalent to clustering with 100% similarity, i.e., amplicon sequence variants (ASVs), was obtained, and sequence length distribution analysis and taxonomic annotation were carried out. There were 1,211,568 pairs of sequences in 16 data samples after sequencing and denoising, with an average of 75,723 pairs of sequences per sample (see Supplementary Table 1). The sequence length ranged from 50 to 432 bp, with an average of 410.83 bp (see Supplementary Table 2). The average number of ASVs in the taxonomic annotation of each sample was 1,380.81 (see Supplementary Table 3). QIIME2 software was used for taxonomic composition analysis, plotting, and computing the alpha diversity index. An R script was adopted to conduct non-metric multidimensional scaling (NMDS) analysis and between-group analysis of the weighted UniFrac distance matrix. An R script was also used to calculate the principal component coordinate scores and load values for each sample and its genus level and to generate an orthogonal projections to latent structures discriminant analysis (OPLS-DA) plot and a loading plot.

### Treatment Methods

In the medicated group, MG patients were given Fufang Huangqi Decoction (provided by the pharmacy at the Affiliated Hospital of Liaoning University of Traditional Chinese Medicine), which was taken orally before breakfast and after dinner (100 ml/dose).

### Efficacy Evaluation Methods

The absolute and relative scoreof MG (ARS–MG) (19) was used as the evaluation criterion for medication efficacy. Relative score = (absolute score before medication – absolute score after medication) ÷ absolute score before medication × 100%. Patients with a relative score >25% were selected to represent patients whose symptoms improved significantly.

### Statistical Analysis

The gut microbiota data were analyzed by ASV clustering and taxonomic analysis, and all the data were analyzed and processed using SPSS 24.0 statistical software. Count data are presented as rates, and the χ^2^ test was used for comparisons between the groups. Measurement data are presented as the mean ± standard deviation ($\bar{x}$ ± *s*). The independent-sample *t*-test was used to compare data with a normal distribution and homogeneous variance; the Mann–Whitney U test was adopted for data that did not conform to a normal distribution. Alpha diversity indices were analyzed using QIIME2 (2019.4) and R analytics. NMDS analysis was performed using R analytics with a weighted UniFrac distance algorithm and an elliptical confidence level of 0.95, with a stress <0.2 indicating that the graph has some explanatory significance. Between-group differences were analyzed using the analysis of similarities (ANOSIM) test with a weighted UniFrac distance algorithm. OPLS–DA analysis was performed using R analytics with an elliptical confidence level of 0.95. *P* < 0.05 indicates a statistically significant difference.

## Results

### Alpha Diversity Analysis, Beta Diversity Analysis (NMDS and Intergroup Difference Analysis)

**Figure 1:**
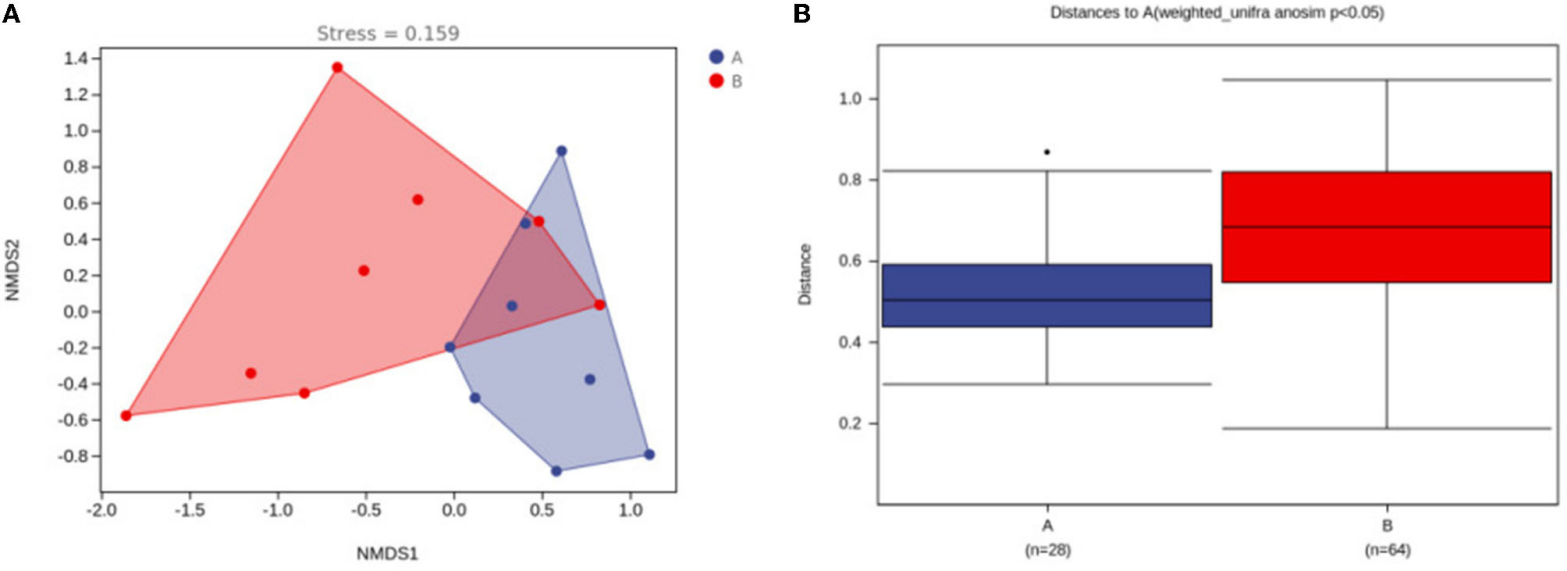
Differences in microbial communities between the control group and the medicated group. **(A)** NMDS analysis between the 2 groups; **(B)** analysis of differences between the 2 groups. A, control group; B, medicated group.

To explore the diversity of the microbiota in the control group and the medicated group within and between habitats, the alpha diversity and beta diversity of both groups were analyzed. In terms of alpha diversity analysis, among alpha diversity indices, we analyzed the Chao1 index, the observed species index, the Shannon index, Faith's PD index and Good's coverage index. The Chao1 index and the observed-species index characterize richness; the Shannon index characterizes diversity; Faith's PD index characterizes genetic diversity; and Good's coverage index characterizes coverage. We found that the richness, diversity and degree of genetic diversity of the community decreased and the coverage increased in the medicated group compared to the control group, but there was no statistically significant difference (*P* > 0.05) (see Supplementary Figure 1). In the beta diversity analysis, we found that the communities of microorganisms differed more significantly in the NMDS1 dimension between the control and the medicated groups in the NMDS analysis (see Figure 1A), and the intergroup difference analysis indicated that the community composition was significantly different between the control group and the medicated group (*P* < 0.05) (see Figure 1B).

### Gut Microbiota Taxonomic Composition Analysis

**Figure 2:**
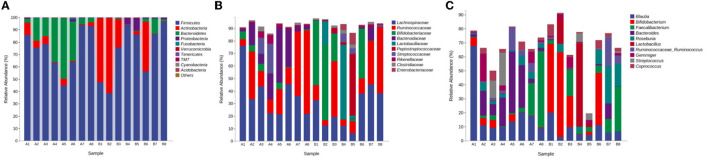
Gut microbiota abundance compositions. **(A)** abundance composition at the phylum level; **(B)** abundance composition at the family level; **(C)** abundance composition at the genus level. A, control group; B, medicated group.

(1) Phylum level: The gut microbiota with the highest average abundance in the control group was Firmicutes (74.74%), followed by Bacteroidetes (20.30%). The gut microbiota with the highest average abundance in the medicated group was Firmicutes (72.67%), followed by Actinobacteria (22.49%). Compared with the control group, the medicated group showed a significantly lower abundance of Bacteroidetes (*P* < 0.05) and a significantly higher abundance of Actinobacteria (see Figure 2A). Compared with that in the control group, the Firmicutes/Bacteroidetes (F/B) ratio in the medicated group was significantly higher (*P* < 0.05). (2) Family level: The gut microbiota with the highest average abundance in the control group was Lachnospiraceae (37.45%), followed by Ruminococcaceae (Firmicutes, 27.24%) and Bacteroidaceae (16.60%). In the medicated group, Lachnospiraceae (25.50%) had the highest average abundance, followed by Bifidobacteriaceae (22.06%) and Ruminococcaceae (21.84%). Compared with the control group, the medicated group had a significantly lower abundance of Bacteroidaceae (*P* < 0.05) and a significantly higher abundance of Bifidobacteriaceae (see Figure 2B). (3) Genus level: The gut microbiota with the highest average abundance in the control group was *Blautia* (19.80%), followed by *Bacteroides* (16.00%). In the medicated group, *Bifidobacterium* (21.45%) had the highest average abundance, followed by *Lactobacillus* (9.98%). Compared with the control group, the medicated group had significantly lower abundances of *Blautia* and *Bacteroides* (*P* < 0.05) and significantly higher abundances of *Bifidobacterium, Lactobacillus* and *Roseburia* (Actinobacteria) (see Figure 2C).

### OPLS–DA

**Figure 3:**
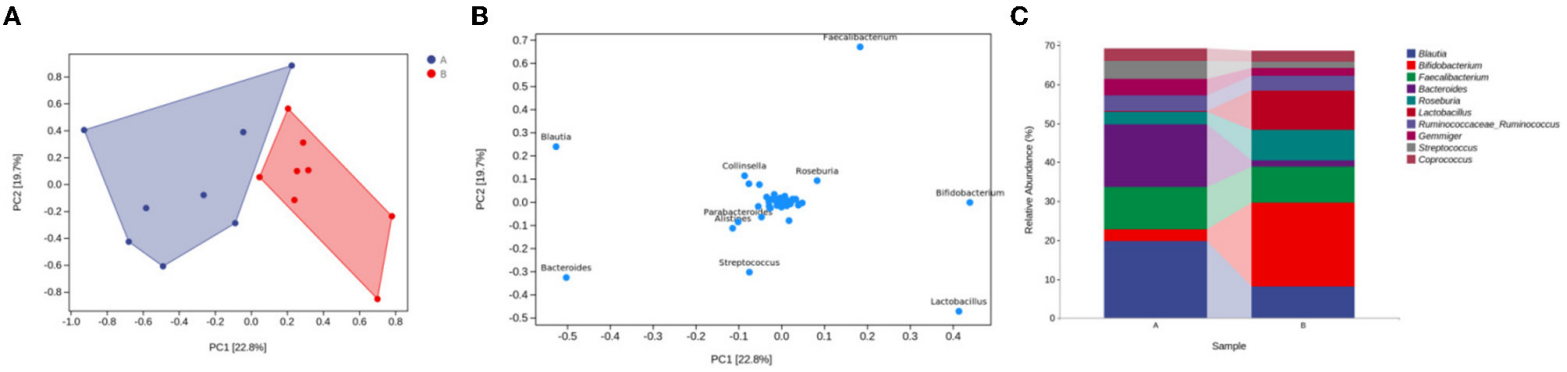
Microbial community composition differences and taxonomic markers in the medicated and control groups. **(A)** OPLS-DA plot for the genus level. **(B)** loading plot for the genus level. **(C)** taxonomic composition analysis of different groups at the genus level. A, control group; B, medicated group.

To determine whether the differences in microbial community compositions between the control group and the medicated group were associated with the different taxonomic distributions, we carried out OPLS–DA at the genus level to obtain an OPLS–DA plot and a loading plot. In the principal component analysis, the interpretation degrees of the PC1 and PC2 dimensions were 22.8 and 19.7%, respectively. The OPLS–DA results indicated that the projection distances between the control group and the medicated group in the PC1 dimension were far away, suggesting that there were obvious differences in taxonomic abundance between the 2 groups (see Figure 3A). The loading plot showed that the variable importance in terms of projection (VIP) values for *Blautia* (52.57%), *Bacteroides* (50.18%), *Bifidobacterium* (43.98%) and *Lactobacillus* (41.43%) were higher in the PC1 dimension. These 4 genera contributed greatly to the differences in the microbial community compositions between the 2 groups (see Figure 3B), as evidenced by the significant differences between the control group and the medicated group in the means of *Blautia, Bacteroides, Bifidobacterium* and *Lactobacillus* in the taxonomic composition analysis at the genus level (see Figure 3C).

## Discussion

The gut microbiota can be divided into symbiotic bacteria, conditionally pathogenic bacteria and pathogenic bacteria based on the relationship with the host. The gut microbiota plays an extremely important role in maintaining the stability of the intestinal environment. An imbalance between symbiotic bacteria and pathogenic bacteria can lead to gut microbiota imbalances. Gut symbiotic bacteria can regulate the mucosal immune system, while pathogenic bacteria can cause immune dysfunction and lead to the occurrence and development of diseases (20). For example, the administration of certain drugs may lead to disturbances in the gut microbiota, which may lead to the occurrence of disease. A national cohort study noted that long-term administration of proton pump inhibitors (PPIs) had an effect on the gut microbiota, which led to changes in the host immune system to induce autoimmune diseases (21). In recent years, multiple studies have shown that the gut microbiota plays a regulatory role in autoimmune diseases (22) and that gut microbiota imbalances may lead to an increase in some inflammatory cytokines and the progression of rheumatoid arthritis (23). *Lactobacillus* may play a preventive role in the pathogenesis of systemic lupus erythematosus; the gut microbiota in a mouse model of systemic lupus erythematosus showed a low proportion of *Lactobacillus* (24). There are some differences in the composition of the gut microbiota between patients with multiple sclerosis and healthy individuals (25). The gut microbiota changes caused by antibiotics may increase the risk of inflammatory bowel disease (26). Moris et al. (14) observed changes in the gut microbiota in healthy controls and MG patients. Compared with healthy controls, MG patients had a higher abundance of Bacteroidetes and a lower abundance of Actinobacteria at the phylum level. This result is consistent with our finding that the medicated group, compared with the control group, had a lower abundance of Bacteroidetes and a higher abundance of Actinobacteria. Recent research has begun to reveal the immune regulation mechanism of the gut microbiota. This regulation is related to some immune cells, and the findings suggest that beneficial gut microbiota can promote immune balance (27, 28). Although the pathogenesis of MG is still not clear, many studies have shown that its pathogenesis is associated with cell-mediated immunity (29, 30). Studies have proposed that specific changes in microbial compositions have a substantial impact on the number of Foxp3+ CD4+ Tregs and the T cell receptor pool and that an imbalance of Foxp3+ CD4+ Tregs may induce the excessive production of AChR antibodies. Foxp3+ CD4+ Tregs can inhibit the production of anti-AChR antibodies, and the pathogenesis of MG is closely related to postsynaptic membrane AChR (31). Therefore, the pathogenesis of MG may have a certain correlation with gut microbiota in immune regulation mechanisms. Fufang Huangqi Decoction has exhibited high efficacy in the treatment of MG. This study explored the effect of Fufang Huangqi Decoction on the gut microbiota in patients with class I or II MG and the correlation between gut microbiota and MG from the perspectives of gut microbiota abundance, diversity within and between habitats, taxonomic differences and corresponding discrimination markers.

The patients who took Fufang Huangqi Decoction and achieved remarkable symptom alleviation had a significantly decreased abundance of Bacteroidetes and significantly increased abundance of Actinobacteria at the phylum level, had a significantly decreased abundance of Bacteroidaceae and significantly increased abundance of Bifidobacteriaceae at the family level and had a significantly decreased abundance of *Blautia* and *Bacteroides* and significantly increased abundance of *Bifidobacterium, Lactobacillus* and *Roseburia* at the genus level. These results highlighted the obviously different gut microbial characteristics in patients with class I or II MG who achieved remarkable symptom alleviation after taking Fufang Huangqi Decoction compared with those who did not take Fufang Huangqi Decoction. The F/B ratio can be used as an indicator of a proinflammatory environment. Proinflammatory microbiota can damage the intestinal epithelium and thereby trigger an immune response, resulting in immune imbalances in autoimmune diseases. Qiu et al. (31) found that the F/B ratio in MG patients was significantly lower than that in a healthy control group. The results were consistent with the decreases in the F/B ratio in inflammatory bowel disease and Crohn's disease. Our results showed that the F/B ratio in MG patients with alleviated symptoms after treatment with Fufang Huangqi Decoction was higher than that in patients who did not take Fufang Huangqi Decoction and that *Blautia* (Firmicutes) and *Bacteroides* were discrimination markers of microbiota, indicating that taking Fufang Huangqi Decoction reduced the proinflammatory microbes and played a regulatory role in the immune imbalance.

We analyzed the alpha diversity in the medicated group and the control group and found no significant differences in abundance, genetic diversity and coverage between the 2 groups. Past studies have indicated that the microbial diversity decreased in some autoimmune diseases but did not decrease in other autoimmune diseases, findings that are consistent with our results. These studies have related the decrease in alpha diversity with disease duration (14), which might also explain why there was no obvious difference in alpha diversity in our results. After taking Fufang Huangqi Decoction for 3–5 years, symptoms in MG patients stabilize. Among the 8 patients in the medicated group whose symptoms were greatly alleviated after taking Fufang Huangqi Decoction, only 1 patient took the medicine for more than 3 years. Therefore, a longer duration of medicine administration may be required to identify the influence of Fufang Huangqi Decoction on gut microbiota diversity.

The beneficial effects of probiotics on hosts are related to gut microbiota balance and the regulation of intestinal and peripheral inflammatory immune responses. Using animal experiments, Consonni et al. (32) evaluated the clinical efficacy of 2 probiotic strains, *Lactobacillus* and *Bifidobacterium*, in the treatment autoimmune diseases such as MG. Their results indicated that these 2 probiotic strains improved the symptoms of experimental autoimmune myasthenia gravis (EAMG) in model rats, reduced the level of serum anti-mouse AChR antibody and increased the content of muscle AChR. Rinaldi et al. (33) showed that the alpha and beta diversity of EAMG gut microbiota changed in rats treated with *Bifidobacterium* and that the relative abundance of *Lactobacillus* was affected by *Bifidobacterium* treatment rather than by inactivated probiotic strains. *Bifidobacterium* and *Lactobacillus* were discrimination markers in our research results and accounted for the significant differences in the gut microbiota between the patients who had obvious symptom alleviation after taking Fufang Huangqi Decoction and patients who did not take Fufang Huangqi Decoction. After taking Fufang Huangqi Decoction, the abundance of *Bifidobacterium* and *Lactobacillus* increased significantly, with a significant alleviation of symptoms in these patients. These studies further evaluated the efficacy of probiotics, especially *Bifidobacterium* and *Lactobacillus*, as an adjuvant therapy for MG. The use of probiotics as complementary therapy for MG has provided a new treatment approach for MG and other autoimmune diseases. However, at present, probiotics as a complementary therapy is only a speculation, and the mechanism of action of probiotics on improving MG is still unclear. In the subsequent trials, we will further explore the relevance of probiotics on MG treatment.

The difference in diet also affects the change in gut microbiota abundance. This study is a real world study, although not uniformly consistent, we explained the need for a light diet to all patients and conducted follow up on diets. In our study, although the diets were slightly different, the overall trend in gut microbiota in the treatment group was significantly better than in the MG patient group.

## Conclusions

In conclusion, Fufang Huangqi Decoction can affect the gut microbiota in patients with class I or II MG, mainly affecting the abundances of *Blautia* (Firmicutes), *Bacteroides, Bifidobacterium* and *Lactobacillus*. Tan et al. (34) found that gut microbial dysbioses are more severe in patients with generalized myasthenia gravis (GMG) than in patients with oculomotor myasthenia gravis (OMG), which is consistent with the clinical manifestations of both MG subtypes. These findings provide us with new ideas for a subsequent large sample study where we will investigate the correlation between different MG subtypes and gut microbiota. Regarding the relationship between the pathogenesis of MG and gut microbiota as well as the use of probiotics as a complementary therapy, it is necessary to further study the influence of gut microbial communities on MG patients and explore the relevant mechanisms.

## Data Availability Statement

The datasets presented in this study can be found in online repositories. The names of the repository/repositories and accession number(s) can be found in the article/Supplementary Material.

## Ethics Statement

The studies involving human participants were reviewed and approved by IRB of the Affiliated Hospital of Liaoning University of Traditional Chinese Medicine. The patients/participants provided their written informed consent to participate in this study.

## Author Contributions

YC performed the experiments in the study and wrote the manuscript. YL, CS, ZL, BX, RH, XH, ZW, JZ, and WQ conceived and designed the research, interpreted the data, and wrote the manuscript. All authors contributed to the article and approved the submitted version.

## Funding

This work was supported by the Key R&D Program of Liaoning Province, China (No. 2020JH2/10300089) and the Construction Project of Liaoning Provincial Key Laboratory, China (No. 2020JH13/10200022).

## Conflict of Interest

BX and RH were employed by the company Zhejiang Jiuru Pharmaceutical Technology Co., Ltd. The remaining authors declare that the research was conducted in the absence of any commercial or financial relationships that could be construed as a potential conflict of interest.

## Publisher's Note

All claims expressed in this article are solely those of the authors and do not necessarily represent those of their affiliated organizations, or those of the publisher, the editors and the reviewers. Any product that may be evaluated in this article, or claim that may be made by its manufacturer, is not guaranteed or endorsed by the publisher.
