# Supplementary material for: Effect of Fufang Huangqi Decoction on the Gut Microbiota in Patients With Class I or II Myasthenia Gravis
Source: Front Neurol. 2022 Mar 18;13:785040. doi: 10.3389/fneur.2022.785040 (PMC8971287; doi:10.3389/fneur.2022.785040)
Supplement: Supplementary file 1 [file Data_Sheet_1.pdf]

Table

Table 1. Statistical table of sequencing amount per sample

SampleID	Input	Filtered	Denoised	Merged	Nonchimeric	Nonsingleton
A1	118697	111716	109648	105911	83252	82817
A2	74793	70090	68370	65037	43526	43144
A3	68944	64608	62630	57569	37328	36680
A4	79506	73832	71913	67320	44215	43626
A5	73119	68588	67013	64067	41608	41152
A6	80008	74682	72627	67878	44607	43831
A7	90755	84451	82551	77804	51495	50996
A8	77252	72808	70974	66626	45723	45187
B1	93172	87913	86536	84080	63368	63084
B2	70618	66725	65595	63843	47738	47551
B3	85813	80134	78262	74408	46467	45977
B4	73287	68590	67067	63459	48300	47765
B5	79318	74809	73225	70025	48530	48124
B6	80261	74258	72026	67015	46039	45362
B7	85707	79871	77970	73525	47095	46645
B8	93069	87492	85161	77941	48576	47736

Table 2. sequence length distribution

length	count	length	count
50	82	407	19480
51	6	408	35693
163	3	409	1892
185	2	410	10987
191	2	411	850
192	2	412	53535
255	3	413	478
261	2	414	32729
273	2	415	189
290	3	416	5
318	5	417	8
338	4	421	29
346	3	422	138
366	2	423	483
368	2	424	1223
379	2	425	78071
380	5	426	717
381	1	427	43
383	2	428	25
395	2	429	6594
404	32533	430	74175
405	395501	431	3855
406	38287	432	212

Table 3. Statistical table of taxonomic annotation results of species

ID	domain	phylum	class	order	family	genus	species	unclassified
A1	8	2	1	251	336	468	124	0
A2	4	0	4	175	411	523	202	0
A3	19	0	4	298	412	544	199	0
A4	43	4	3	271	318	840	243	0
A5	27	1	5	134	230	822	203	0
A6	19	1	1	289	557	758	173	0
A7	8	0	1	225	575	430	247	0
A8	7	1	1	215	578	411	287	0
B1	3	2	1	114	282	346	138	0
B2	10	9	2	109	203	232	206	0
B3	13	5	2	240	468	307	217	0
B4	21	19	3	216	293	393	277	0
B5	34	6	9	146	386	241	190	0
B6	6	4	3	279	627	549	249	0
B7	10	0	2	204	444	581	182	0
B8	6	4	0	269	840	506	272	0

Figure

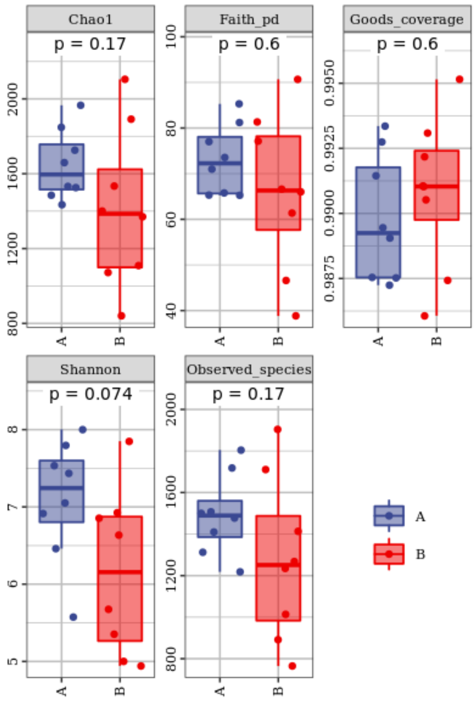

Figure 1. Alpha diversity index. A. control group; B. medicated group

Ref 1.

The Reference of DADA2 Methods:
Callahan BJ, McMurdie PJ, Rosen MJ, Han AW, Johnson AJ, Holmes SP. DADA2: High-resolution sample inference from Illumina amplicon data. *Nat Methods*. (2016) 13: 581-583. doi: 10.1038/nmeth.3869.

Datasets

Registration number, ChiCTR2100048367;

Registration website, http://www.chictr.org.cn/listbycreater.aspx;

Raw data of gut-microbiome, NCBI: SRP338707.
